# Interaction between steady-state visually evoked potentials at nearby flicker frequencies

**DOI:** 10.1038/s41598-020-62180-y

**Published:** 2020-03-24

**Authors:** Siddhesh Salelkar, Supratim Ray

**Affiliations:** 1 IISc Mathematics Initiative, Department of Mathematics, Indian Institute of Science, Bangalore, 560012 India; 2 Centre for Neuroscience, Indian Institute of Science, Bangalore, 560012 India

**Keywords:** Neuroscience, Neural circuits, Visual system

## Abstract

Steady-state visually evoked potential (SSVEP) studies routinely employ simultaneous presentation of two temporally modulated stimuli, with SSVEP amplitude modulations serving to index top-down cognitive processes. However, the nature of SSVEP amplitude modulations as a function of competing temporal frequency (TF) has not been systematically studied, especially in relation to the normalization framework which has been extensively used to explain visual responses to multiple stimuli. We recorded spikes and local field potential (LFP) from the primary visual cortex (V1) as well as EEG from two awake macaque monkeys while they passively fixated plaid stimuli with components counterphasing at different TFs. We observed asymmetric SSVEP response suppression by competing TFs (greater suppression for lower TFs), which further depended on the relative orientations of plaid components. A tuned normalization model, adapted to SSVEP responses, provided a good account of the suppression. Our results provide new insights into processing of temporally modulated visual stimuli.

## Introduction

The steady-state visually evoked potential (SSVEP) is a stimulus-locked oscillatory response to periodic visual stimulation commonly recorded in electroencephalogram (EEG) studies in humans^1^. Due to its high signal-to-noise ratio, relative immunity to artifacts, non-invasiveness and ease of recording, the SSVEP has become a popular modality in visual cognitive neuroscience research^2,3^. When two or more flickering stimuli are simultaneously presented, in addition to the SSVEP and their harmonics, intermodulation (IM) components are also produced due to nonlinear interactions between the neural substrates producing the SSVEP^4^. The SSVEP and IM responses are known to be sensitive to top-down cognitive modulatory effects, and numerous studies have used such paradigms to investigate a wide variety of cognitive and perceptual phenomena^5–12^.

SSVEP has often been used in masking paradigms, in which human observers detect a temporally modulated target grating when it is superimposed by another temporally modulated mask grating presented at different contrasts and orientations relative to the target^13–16^. These studies have shown that the target SSVEP is reliably suppressed in the presence of the mask, depending on its contrast and relative orientation. Masking suppression has been shown to be broadly tuned^14,17^, which is consistent with literature from neurophysiology showing that inhibition in single neurons is also broadly tuned, with similar orientations causing stronger suppression than dissimilar orientations^18–20^. Previous reports^13,21,22^ suggest that masking could be temporally asymmetric such that lower mask frequencies could behave differently than higher mask frequencies, but this has not been tested using invasive recordings.

In the context of sensory processing, presentation of two stimuli within a visual neuron’s receptive field is known to cause marked differences in the spiking response as compared to either stimulus presented alone; this has been explained using normalization, which dictates how a neuron combines stimuli in its receptive field^23–25^ such that the response to a combination of preferred and non-preferred stimuli is usually weaker than the response to the preferred alone. Normalization is thought to operate throughout the visual cortical hierarchy as well as across modalities^26^. Unsurprisingly, masking studies have also used variants of the normalization model to explain SSVEP suppression^15–17^. Normalization models in the neurophysiology literature commonly explain the aggregate response of a cell or neural population in a given time window^23,25,27^, but a few recent studies have also explored more dynamic formulations^28,29^. Masking studies have usually employed temporal domain models, which can also account for IM responses across different stimulus conditions^15–17^. Notwithstanding, a common feature of all these normalization models is the “normalization pool”, which is broadly tuned for stimulus attributes (such as orientation and spatial frequency) and accounts for the experimentally observed suppression by appearing as a divisive term in the denominator of the model^17,25^. It is plausible that the normalization pool is also tuned to temporal frequency^30^, but how this shapes target SSVEP response suppression as a function of the mask frequency is presently unknown.

Traditionally, most SSVEP studies have been performed using human EEG. Despite the widespread use of SSVEP, its precise mechanisms remain unclear, although the dominant view holds that the SSVEP results from the synchronized activity of spatially homogeneous cortical neurons in an open-field arrangement^31^. This is a simplified assumption, however, since the surface EEG can relate to the underlying neural activity in more complex ways^32–35^. Attempts to probe the nature of TF normalization at multiple scales of neural recording, therefore, could potentially shed light on the mechanisms of SSVEP generation as well.

To address these questions, we recorded spiking activity and local field potential (LFP) from the primary visual cortex (V1) as well as scalp EEG, from two awake, fixating female macaque monkeys while they viewed sinusoidal gratings and plaids, and analyzed the modulation of SSVEP response as a function of the driving stimulus frequencies. We presented one of the gratings of the plaid at either 8 Hz or 16 Hz (since SSVEP amplitude response has generally been observed to be the largest in this range) while varying the TF and orientation of the second grating relative to the TF and orientation of the first. To explain the results, we adapted the normalization model to SSVEP responses and characterized the nature of suppression as a function of the difference in TFs and orientations used.

## Results

We recorded spiking activity and LFP using chronic arrays consisting of 96 microelectrodes (Blackrock Microsystems) implanted in the right hemisphere of V1, along with simultaneous surface EEG from up to 7 electrodes. The monkeys passively fixated within a 2° window around a fixation spot at the center of the screen while full-field stimuli (gratings and plaids in various configurations; see Methods for details) were presented for 1500 ms each (2 stimuli per trial, with an interstimulus interval of 1500 ms).

### Spikes, LFP and EEG have different tuning for preferred TF

**Figure 1:**
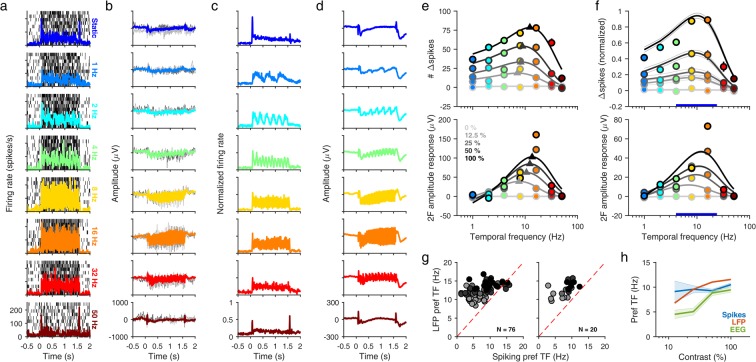
SSVEP tuning of MUA, LFP and EEG. **(a)** Raster plots (thin black ticks) and peri-stimulus time histogram (PSTH, solid colored trace) plots of the MUA response of a typical electrode (Monkey 1, electrode 2) to a full-contrast 90° sinusoidal grating presented at a range of counterphase frequencies in Experiment 1. Rows show TF conditions from top to bottom: static, 1 Hz, 2 Hz, 4 Hz, 8 Hz, 16 Hz, 32 Hz and 50 Hz. **(b)** Evoked response (solid colored trace) for the same electrode and session. Two example trials (thin light and dark gray traces) are also plotted for comparison. **(c)** PSTH plots showing mean firing rate for the entire population of MUA electrodes (N = 32), normalized across sessions. MUA electrodes were selected from LFP electrodes having a SNR of ≥2, which had accumulated ≥2000 spikes during the recording, and showed a firing rate increase of ≥5 spikes/s (Monkey 1) or ≥3 spikes/s (Monkey 2) in the 0.5–1.5 sec period after stimulus onset for the full contrast, static grating. **(d)** Mean evoked response for the entire population of LFP electrodes (N = 650) across sessions. **(e)** Difference of exponentials fits for the spiking response (top) and LFP response (bottom) of example electrode shown in (**a**,**b**) respectively, for five contrasts (0, 12.5, 25, 50 and 100%). Fits (grayscale curves) were computed for mean change in spike counts and mean change in SSVEP amplitude at 2F (colored circles) as a function of TF for each contrast separately. Triangles denote the estimated preferred TF for each nonzero contrast. Error bars denote ±1 SEM and are smaller than marker size when not visible. Changes in spike counts and SSVEP amplitudes were computed during 0.5–1.5 sec after stimulus onset, using the 1 sec pre-stimulus period as baseline. **(f)** Difference of exponentials fits for the normalized MUA response (top) and LFP response (bottom) across the entire population of MUA (N = 32) and LFP (N = 650) electrodes respectively. Error bars/curves denote ±1 SEM. Thick blue bar indicates the combined TF range over which mask frequency was varied in Experiment 2. **(g)** Scatter plots of preferred TFs estimated for MUA (x-axis) and LFP (y-axis) responses from the same electrode. Each data point represents an electrode having fit quality ≥0.75 and preferred TF ≥ 1 Hz for both MUA and LFP responses for a contrast condition (gray level). Left panel, all electrodes meeting chosen criteria. Right panel, spiking electrodes above cutoffs chosen in (**c**). **(h)** Mean preferred TF as a function of increasing contrast, estimated from band-pass (preferred TF ≥ 1 Hz) difference of exponentials fits for MUA, LFP and EEG (N = 4) electrodes. Shaded error bars denote ±1 SEM.

In Experiment 1, we measured SSVEP responses to counterphase gratings presented at a range of contrasts (n = 5; 0, 12.5, 25, 50, 100%) and TFs (n = 8; 0, 1, 2, 4, 8, 16, 32, 50 Hz) while varying orientations (6 orientations, uniformly spaced between 0–150°) across sessions. This allowed us to obtain an estimate of SSVEP response tuning and compare it with other response measures. Figure 1a,b show the spiking and LFP responses of a typical electrode at full contrast (Monkey 1, electrode 2; 90° counterphase grating presented at 8 TFs). Gratings at lower temporal frequencies induced higher frequency oscillations (gamma band responses at ~30–70 Hz) in the LFP signal in addition to SSVEP responses; however, stimuli at higher temporal frequencies produced significant SSVEP responses but almost negligible gamma oscillations (Supplementary Fig. S1). As expected for counterphase stimulation, there was a prominent SSVEP response at double the stimulation frequency (2F), which was also visible when averaged across the population of spiking electrodes (Fig. 1c; electrodes were selected from each session using predetermined cutoffs before being pooled together, see Methods; n = 32; Monkey 1: n = 20; Monkey 2: n = 12) and LFP electrodes (Fig. 1d; electrodes having consistent LFP responses and stable estimates of receptive field across days; n = 650; Monkey 1: n = 65 electrodes × 7 sets across 6 orientations; Monkey 2: n = 39 electrodes × 5 sets across 4 orientations) at full contrast. The SSVEP responses could also be observed in simultaneously recorded occipital EEG electrodes (Supplementary Fig. S2).

Previous studies have shown that TF tuning is not invariant with contrast; the preferred TF increases as contrast increases^23,24^. To estimate the TF tuning of different SSVEP response measures in our experiment, we fit a difference-of-exponentials function^36,37^ to the response measure for each contrast condition. For spiking electrodes, we fit the mean difference in spike counts from baseline (Fig. 1e, top; fit to example spiking responses from the same electrode and session as in Fig. 1a). For LFP electrodes, we used the mean difference in SSVEP amplitude (in μV) from baseline (Fig. 1e, bottom; fit to LFP amplitude responses from the same electrode in Fig. 1b but averaged across sessions; also see Supplementary Fig. S2 for fit to EEG amplitude responses averaged across sessions for occipital electrodes from both monkeys). Fit quality was quantified as in our earlier report^37^; for all three response measures, we generally obtained good fits to data across all non-zero contrast conditions in both monkeys (Supplementary Fig. S2).

Consistent with previous studies, the preferred TF increased with stimulus contrast for both spiking (Fig. 1f, top; n = 32) and LFP responses (Fig. 1f, bottom; n = 650). We observed some notable differences between LFP and spike response tuning. The fits for many spiking electrodes were low pass at low contrasts and became band-pass at higher contrasts. Most LFP electrodes, on the other hand, showed good band-pass fits even at the low contrasts in our experiment. LFP responses peaked around the upper alpha (~12–16 Hz) range but were more closely clustered (that is, more similar in their tuning), whereas spiking responses tended to prefer slightly lower TFs than LFP responses but spanned a wider range (Supplementary Fig. S2). LFP responses showed a sharp increase at ~16 Hz as compared to other TFs, which was more prominent at higher contrasts and was evident in the fits deviating quite a bit from the data points (Fig. 1f, bottom; orange circles, 50% and 100% contrasts). Notably, spiking responses did not show such behavior (Fig. 1f, top). As an additional measure, we directly compared spiking and LFP tuning obtained from the same electrode, in a subset of electrodes which showed good fits to both spiking and LFP data for at least one contrast condition in at least one session (see Methods). We saw similar tuning behavior as above; the preferred LFP TF was significantly higher than the preferred spiking TF for most of the electrodes (Fig. 1g; *p* < 0.001 in both cases; paired Wilcoxon signed-rank test, one-tailed) which was clearly visible at higher contrasts (Fig. 1h).

EEG electrodes (occipital electrodes from each monkey) showed similar contrast tuning as spiking and LFP responses but appeared to prefer lower TFs (Fig. 1h). We interpret the EEG tuning results with caution since the SSVEP responses were often noisier, especially at lower contrasts, due to a small number of trials.

### Competing TFs can potentially interact in multiple ways

In Experiment 2, we sought to characterize how the presence of two competing TFs in the visual field can have interactional effects on their SSVEP amplitudes. To this end, we measured SSVEP responses to plaids formed by the superposition of two gratings counterphasing at different frequencies, while the monkeys passively fixated. To check whether the orientations of the gratings have any bearing on TF interactions, we also varied the relative orientation difference between the two gratings (parallel, 30° separation, 60° separation, orthogonal) across sessions. Since SSVEP response amplitudes in spiking and LFP were found to peak around the 8–16 Hz range (Fig. 1), we presented one of the gratings at either 8 Hz or 16 Hz (“target” frequency, f_TARGET_, separate sessions for each), while the TF of the other grating (“mask” frequency, f_MASK_) varied in a close range around f_TARGET_ between lower delta (2 Hz) to high beta (30 Hz), depending on the f_TARGET_ used in that session. The target grating was always presented at a fixed contrast of 25%, whereas the mask grating was presented at either 0% or 25% contrast, allowing us to measure grating and plaid SSVEP amplitudes in the same experiment. Note that there was no behavioral task of detecting the target in the presence of the mask in our experiments; we mainly use the terminology of “target” and “mask” for clarity.

**Figure 2:**
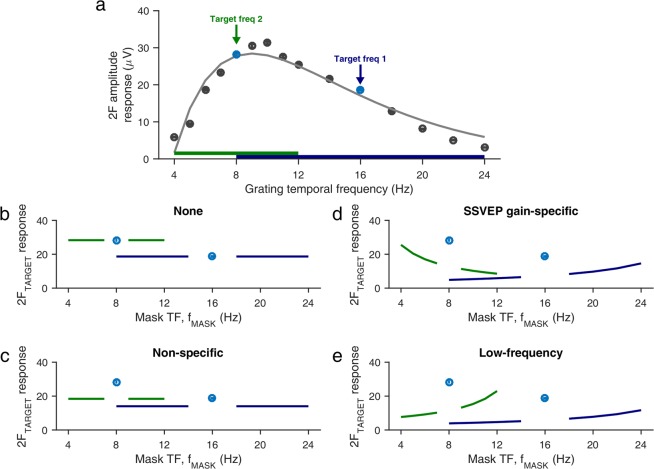
Hypotheses for interaction between competing SSVEP tags. Mean SSVEP amplitude response over LFP electrodes from both monkeys as a function of TF **(a)**, averaged over 25% contrast grating conditions from Experiments 1 and 2. Error bars denote ±1 SEM and are smaller than the marker size when not visible. Smooth gray curve indicates the difference of exponentials fit to the mean response. Arrows and thick horizontal bars indicate, respectively, the target frequencies and the corresponding mask frequencies used in Experiment 2. Panels below depict hypothetical target frequency SSVEP amplitude responses for no TF suppression **(b)**, non-specific suppression **(c)**, SSVEP gain-specific suppression **(d)** and low-frequency suppression **(e)**.

Figure 2a depicts the TF tuning of a grating at 25% contrast, and we analyzed how the amplitude at 2F_TARGET_ (Fig. 2a, arrows) varies as a function of f_MASK_ (Fig. 2a, thick horizontal bars). Several candidate hypotheses are shown in Fig. 2b–e. The simplest possibility is the absence of any TF normalization; the target SSVEP amplitude is unaffected by f_MASK_, so that the plaid target amplitude is identical to the grating target amplitude (Hypothesis 1, Fig. 2b). Previous masking studies^13,14,17^ show that this is not the case. A second possibility is non-specific or symmetric suppression, in which the plaid target amplitude is suppressed relative to the grating target amplitude, but the magnitude of suppression is essentially independent of f_MASK_ (Hypothesis 2, Fig. 2c). The magnitude of suppression may be frequency-tuned so that the suppression is different for 16 Hz and 8 Hz target frequencies.

Finally, the magnitude of TF suppression may be asymmetric and depend on f_MASK_ relative to f_TARGET_. One possibility is that the strength of TF suppression may be SSVEP gain-specific, or in other words, tuned to absolute TF, such that the magnitude of suppression is proportional to the SSVEP tuning strength of f_MASK_ (Hypothesis 3, Fig. 2d). In other words, if this hypothesis were true, we would expect to see maximum suppression at ~10–12 Hz where the relative increase in SSVEP amplitudes from baseline were the largest (Fig. 2a). This also means that we would see the 16 Hz target amplitude getting suppressed more by f_MASK_ < 16 Hz, whereas the 8 Hz target amplitude would be suppressed more by f_MASK_ > 8 Hz.

An alternate possibility is the normalization strength depends not on the relative increase in power from pre-stimulus baseline (as shown in Fig. 2a) but instead on the absolute power. Due to the *1/f*^* n*^ nature of the power spectral density (PSD) of the LFP and EEG, this would mean that lower frequencies that have much greater absolute power than higher frequencies would be more suppressive, such that mask frequencies lower than the target frequency would suppress the target frequency more than mask frequencies higher than the target frequency (Hypothesis 4, Fig. 2e). There could be other reasons for such a scenario as well. For example, if the normalization signal is generated by integrating the pooled responses over some temporal window (effective low-pass filtering), higher mask TFs will contribute less to the normalization pool than low TFs^16^. In such a case, the nature of suppression would be similar for the 16 Hz and 8 Hz target frequencies. Note that given the way we have formulated our hypotheses, the behavior of SSVEP amplitude modulation in the 8 Hz target frequency is crucial for differentiating between Hypotheses 3 and 4, since the amplitude modulation in the 16 Hz target frequency is similar in both cases.

### Suppression depends on both relative orientation and TF

**Figure 3:**
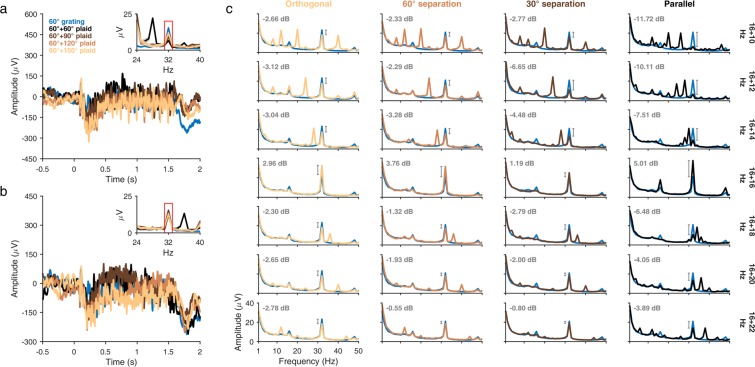
LFP amplitude suppression (16 Hz target frequency). **(a)** Evoked response of a typical LFP electrode (Monkey 1, electrode 90) to plaid stimuli composed of a 16 Hz target grating (60°) and a 12 Hz mask grating (60°, black; 90°, brown; 120°, orange; 150°, light brown), all recorded in the same session. Amplitude spectrum of the 16 Hz mask-only condition (60°, blue) is plotted for comparison. Inset shows the LFP amplitude spectra highlighting the target SSVEP (red rectangle). **(b)** Evoked response of the same LFP electrode with the mask grating contrast-reversing at 20 Hz, other stimulus conditions remaining the same. Inset shows the corresponding LFP amplitude spectrum. **(c)** Mean amplitude spectrum of the population of LFP electrodes across both monkeys for the 16 Hz target frequency, with rows showing different TF conditions and columns showing different orientation conditions. Target-only amplitude spectrum (blue) is averaged across sessions from Experiment 1 for the 16 Hz, 25% contrast condition. Numbers indicate the mean change in power (in dB) at 32 Hz from the target-only condition to the corresponding plaid conditions.

We first studied how the 16 Hz SSVEP target frequency amplitude in the LFP changes as a function of the competing mask frequency and orientation (see Methods). Figure 3a,b show the evoked response for an example LFP electrode (Monkey 1, electrode 90) for the 12 Hz and 20 Hz mask frequencies, respectively, from a session in which all four orientation conditions were recorded on the same day for the 16 Hz target frequency orientation of 60°. Interestingly, the LFP amplitude spectrum showed a higher suppression of the target SSVEP amplitude (at 2F_TARGET_ = 32 Hz) for the 12 Hz f_MASK_ than for the 20 Hz f_MASK_ and varied as a function of the relative orientation difference between the target and mask (compare Fig. 3a,b insets).

To determine whether this was true for our population of LFP electrodes, we averaged the spectra for all LFP electrodes across all 16 Hz target frequency sessions from both monkeys and compared the 2F_TARGET_ SSVEP amplitude modulations between different conditions (Fig. 3c). We observed that in all orientation conditions (Fig. 3c, columns; left to right, orthogonal to parallel), there was a reliable suppression of the 16 Hz target frequency SSVEP amplitude by all nearby mask frequencies (gray annotations within panels), indicating a robust presence of TF normalization and suggesting that Hypothesis 1 (Fig. 2b) can be ruled out as expected. For the orthogonal plaid (Fig. 3c, leftmost column), the suppression was approximately symmetric, with mask frequencies both above and below 16 Hz almost equally suppressing the target SSVEP, which seems to be consistent with Hypothesis 2 (Fig. 2c). However, as the relative orientation difference between the two component gratings decreased and the gratings became more and more similar in their orientation (Fig. 3c, second-left to rightmost columns), the degree of asymmetry in the suppression increased; mask frequencies less than 16 Hz more strongly suppressed the target SSVEP than mask frequencies greater than 16 Hz. The pattern of suppression, now, seemed to be consistent with Hypothesis 3 (Fig. 2d) or Hypothesis 4 (Fig. 2e). These results suggest that the nature of TF suppression is not fixed, but rather is governed by the relative orientations and TFs of the gratings generating the SSVEP. Note that there were additional peaks in the PSDs corresponding to sub-harmonics and IM components. For example, we found a peak at F_TARGET_ + F_MASK_ for the parallel case, but not at any other condition, consistent with a prior finding^15^ that the F_TARGET_ + F_MASK_ peak is salient only when the target and mask orientations are close to each other. For the non-parallel conditions, a prominent peak was instead observed at |2F_TARGET_ − 2F_MASK_ | . We discuss the IM components in more detail later.

### TF suppression is low frequency tuned

**Figure 4:**
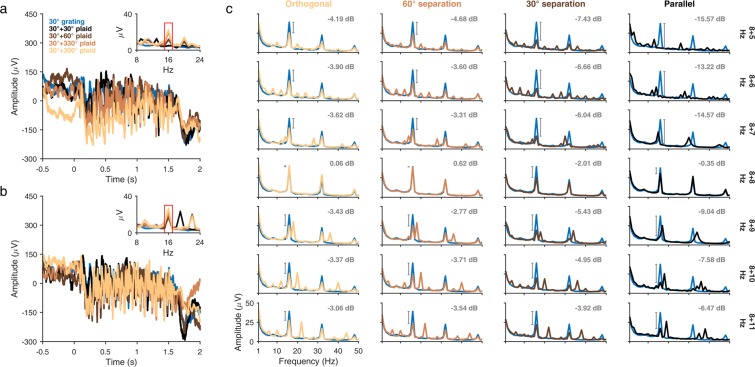
LFP amplitude suppression (8 Hz target frequency). Same as in Fig. 3, but for the 8 Hz target frequency. Evoked response is plotted for the same monkey and electrode for plaid stimuli composed of an 8 Hz target grating (30°) and a 5 Hz **(a)** or 11 Hz **(b)** mask grating (30°, black; 60°, brown; 330°, orange; 300°, light brown). Amplitude spectrum of the 8 Hz mask-only condition (30°, blue) is also plotted. Target-only amplitude spectrum in **(c)** is averaged across sessions from Experiment 1 for the 8 Hz, 25% contrast condition. Numbers in (**c**) indicate mean change in power (in dB) at 16 Hz from the target-only condition to the corresponding plaid conditions.

To distinguish between Hypothesis 3 and Hypothesis 4, we next looked at the corresponding 2F_TARGET_ SSVEP amplitude modulation patterns for the 8 Hz target frequency (Fig. 4). Here, we used the same number of mask frequencies as for the 16 Hz target frequency but spaced evenly in steps of 1 Hz from the 8 Hz target.

Results were broadly similar to the ones for the 16 Hz target frequency, as shown for the same example LFP electrode (Fig. 4a, 8 + 5 Hz; Fig. 4b, 8 + 11 Hz). For the orthogonal condition (Fig. 4c, leftmost column), we observed nearly symmetric suppression by mask frequencies both above and below 8 Hz, which became strongly asymmetric as the orientations became parallel (Fig. 4c, rightmost column) with lower mask frequencies more strongly suppressing the 8 Hz target SSVEP than higher mask frequencies. This observation, together with the results for the 16 Hz target frequency (Fig. 3), suggest that suppression in the V1 SSVEP is “tuned” in the joint orientation and TF space: mask frequencies lower than the target are more suppressive compared to mask frequencies greater than the target, and the degree of this asymmetric suppression depends on the orientation of the mask relative to the target.

**Figure 5:**
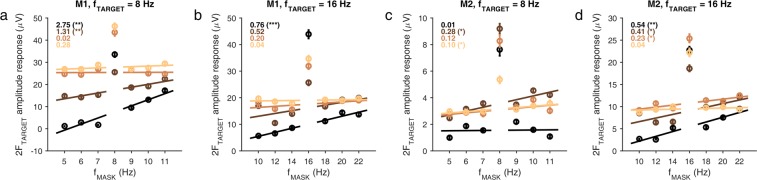
SSVEP amplitude suppression summary (individual monkeys). Plots showing summary of amplitude suppression in LFP as a function of relative orientation and mask TF for the two monkeys [f_TARGET_ = 8 Hz, **(a,c)**; f_TARGET_ = 16 Hz, **(b,d)**]. Colors denote relative orientation as in Figs. 3 and 4. Error bars indicate ±1 SEM across electrodes. Lines show the fits of linear regression across the six data points (three below and three above the target frequency). The mean slopes and their associated significance levels (***p < 0.001; **p < 0.01; *p < 0.05) are shown on top left corner.

Figure 5 shows the summary of the results separately for each monkey. The asymmetry was quantified by fitting a line to the mean SSVEP amplitude responses at 2F_TARGET_ (16 Hz for f_TARGET_ = 8 Hz, Fig. 5a,c; 32 Hz for f_TARGET_ = 16 Hz, Fig. 5b,d) for the six masking frequencies (three below and three above each target frequency; we also fitted the lines separately to the points either below or above the target frequency, but the fits were noisier because this approach led to only three data points for each fit). In general, slopes were close to zero for orthogonal orientations, tended to increase with decreasing difference between orientations and were largest for parallel orientations. One exception was the parallel condition at 8 Hz for Monkey 2 (Fig. 5c, black trace), which was almost flat. Some of these monkey-specific differences are discussed later. EEG slopes were comparable to, but noisier than, the corresponding LFP slopes for both monkeys (Supplementary Fig. S3).

Many studies have used IM components to study stimulus interactions^10,11,15,16,38^. When we analyzed the IM components at 2|F_TARGET_ − F_MASK_ | using a similar approach as above, we did not observe more suppression at low masking frequencies, but instead observed inconsistent suppression for mask frequencies very close to the target (Supplementary Fig. S4). However, this happens because when target and mask frequencies are very close to each other, the IM responses are generated at very low frequencies (for example, at 2 Hz for f_TARGET_ = 8 Hz and f_MASK_ = 9 Hz), which cannot be estimated clearly because the LFP power is already very high at low frequencies (because of the *1/f*^* n*^ power law).

### Plaid stimuli generate beat modulation of spiking activity

Since our focus was on characterizing the modulation of SSVEP amplitudes in LFP and EEG, the experiments and stimuli were neither explicitly designed for eliciting spiking activity nor tuned to preferred orientations or receptive field sizes of our spiking electrodes. In particular, we used full screen stimuli to maximize the SSVEP response in the EEG, which, as in our previous studies^39,40^, led to large suppression of firing rates. Consequently, reliable MUA could be recorded from only a few electrodes in Experiment 2, as described below.

**Figure 6:**
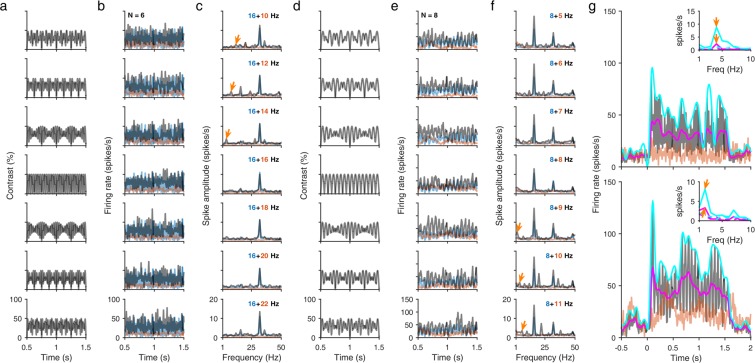
Beat modulation of MUA to orthogonal plaid stimuli. **(a,d)** Stimulus contrast profiles for orthogonal plaid stimulus as a function of mask frequency (rows) for the 16 Hz target frequency (**a**) and 8 Hz target frequency (**d**). Target and mask frequencies were full rectified before being added to generate the contrast profiles (see Methods). **(b**,**e)** PSTH plots showing the mean MUA across selected spiking electrodes for the grating (blue, target-only; orange, mask-only) and orthogonal plaid (black) stimuli for the 16 Hz target frequency (N = 6, b) and 8 Hz target frequency (N = 8, e) in Experiment 2. **(c**,**f)** Mean MUA spectra for the PSTH data shown in (**b**,**e**) respectively. Inset labels denote the TFs of the plaid stimuli. Arrows (gold) label |2F_TARGET_ − 2F_MASK_ | IM components. **(g)** Magnified PSTH traces for the 16 + 14 Hz plaid (top) and 8 + 9 Hz plaid (bottom) showing the beat modulation of the envelope (cyan) and the moving average (magenta) of the MUA. Insets show the corresponding spectra with the IM components (arrows).

We first studied well-tuned MUA when a plaid composed of the preferred (target) and null (mask) orientations were presented (Fig. 6). Target frequency gratings, when presented alone, elicited robust firing (blue; Fig. 6b, 16 Hz, n = 6; Fig. 6e, 8 Hz, n = 8), observed as prominent peaks at 2F_TARGET_ in the corresponding firing rate spectra (blue; Fig. 6c,f). The orthogonal grating presented on its own elicited only a weak or no response (orange; Fig. 6b,e) with inconspicuous peaks in the firing spectra (orange; Fig. 6c,f). However, for both target frequencies, there was a significant temporal modulation of firing rates with the superimposition of the two gratings to form a plaid (black; Fig. 6b,e), producing small but noticeable peaks at the IM components (gold arrows; Fig. 6c,f). This temporal modulation resembled the IM components that could be observed in the contrast profiles of the plaids due to full wave rectification (Fig. 6a,d; see Methods). To observe this more clearly, one condition for each target frequency is plotted (Fig. 6g; top, 16 + 14 Hz; bottom, 8 + 9 Hz) along with a moving average of the PSTH (magenta trace) as well as the envelope (cyan trace). Both show modulation at the ‘beat’ frequency (|2F_TARGET_ − 2F_MASK_|, or 4 and 2 Hz for the top and bottom conditions), which are also observed in their spectra (Fig. 6g, insets). Thus, although we did not have sufficient number of MUA for statistical analysis, the clear ‘beat’ modulation of MUA activity for orthogonal plaids, together with robust spiking for the preferred grating and little or no spiking for the orthogonal grating, suggests the involvement of orientation-based normalization mechanisms underlying the observed time course of neuronal spiking responses, reminiscent of the results of an earlier study^18^.

We also looked at spike spectra for all MUA with appreciable firing rates (see Methods for details), for different orientations of target and mask TFs, in a format similar to Figs. 3 and 4 (see Supplementary Figs. S5 and S6). Spikes were clearly locked to the temporal frequencies and produced distinct peaks at the target and mask frequencies as well as salient IM components. However, the pattern of asymmetry observed in the LFP SSVEPs, especially for parallel orientations, was not observed in these plots. We interpret these results with caution because the stimuli used here were not optimal for spiking (leading to very few MUA units) and these plots were averaged over conditions that were not always recorded in the same session. Nevertheless, these potentially interesting differences between MUA and LFP/EEG SSVEP suppression point to different mechanisms by which normalization may act across spatial scales, as discussed later.

### A tuned normalization model can explain the size of SSVEP amplitude modulations

To explain our results, we used a variant of the “tuned” normalization model that has been recently used in the context of primate visual cortex^41–44^, adapting it from an earlier model which explained simple cell responses in macaque V1^23^. We fit the data from Experiments 1 and 2 separately, in two steps.

**Figure 7:**
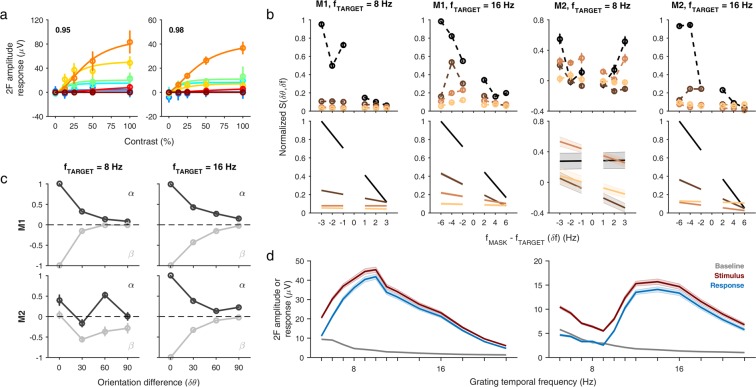
A tuned normalization model explains SSVEP suppression effects. **(a)** Fits of the model (Eqs. 1 and 2) to the mean LFP amplitude responses (circles) for two representative electrodes (left, Monkey 1, electrode 90; right, Monkey 2, electrode 65) from Experiment 1. Colors indicate grating TF as in Fig. 1. Inset numbers indicate the fraction of variance in the data captured by the fit. Error bars indicate ±1 SEM across sessions. **(b)** Empirical suppression values (circles and dotted traces, top row) and fitted suppression values (solid lines, bottom row) obtained from the normalization model (Eqs. 3 and 4) using LFP amplitude responses for the 8 Hz and 16 Hz target frequencies in Experiment 2 for each monkey. Colors in each panel indicate different orientation conditions as in Figs. 3, 4 and 5. Data was normalized before averaging across electrodes for each monkey. Error bars in top row and shaded regions in bottom row indicate ±1 SEM across electrodes for each monkey. **(c)** Fitted parameters α (dark gray) and β (light gray) from the normalization model (Eqs. 3 and 4) as a function of relative orientation for the 8 Hz (left column) and the 16 Hz (right column) target frequencies in the LFP data for the two monkeys (rows). Data was normalized before averaging across electrodes for each monkey. Error bars indicate ±1 SEM across electrodes for each monkey. **(d)** Baseline LFP amplitude (gray), stimulus LFP amplitude (red) and LFP amplitude response (blue) for the two monkeys (left, Monkey 1; right, Monkey 2) around the 8 Hz and 16 Hz target frequencies. Shaded error bars indicate ±1 SEM across electrodes for each monkey.

First, we fit mean SSVEP responses from Experiment 1 to a normalization model for grating responses [Eqs. (1) and (2)], allowing us to estimate model parameters for amplitude, time constants and exponent (see Methods). Figure 7a shows the model fit for the LFP responses of typical electrodes in the two monkeys (left, Monkey 1, electrode 90; right, Monkey 2, electrode 65). Most LFP electrodes showed prominent saturation at mid-level contrasts for lower TFs, and the contrast at which responses saturated increased with increasing TF. We obtained reasonably good fits for spike electrodes (although there was little or no evidence of response saturation at higher contrasts for most TFs) and occipital EEG electrodes, and the fit quality across different response measures was comparable (see Supplementary Fig. S7).

To explain target frequency SSVEP responses as a function of suppression arising from the mask frequency in Experiment 2, we adapted the plaid normalization model^23^ [Eq. (3)], using the term S(δθ, δf) in the denominator of the normalization equation to capture suppression in the joint orientation and TF space (see Methods). Under the assumptions of this model, it was possible to obtain empirical estimates of S(δθ, δf) for different plaid orientation conditions for each target frequency separately in both monkeys (Fig. 7b, top). From these, we modeled and fit S(δθ, δf) as a function linear in the difference between the target and mask frequencies [Fig. 7b, bottom; Eq. (4), parameters α and β]. The mean strength of target suppression across mask frequencies, as measured by α, decreased as δθ increased (Fig. 7c, dark gray), with high values of α for similar orientations and low values of α (although still significantly greater than zero) for dissimilar orientations. More importantly, the dependence of suppression on the difference between target and mask frequencies, captured by β, shows that the asymmetry in the suppression also decreased as δθ increased (Fig. 7c, light gray). Specifically, at the orthogonal orientations (δθ = 90°), the mean β value was almost close to 0, indicating that the suppression is essentially independent of δf. The only exception, as before, was the parallel condition for 8 Hz in Monkey 2.

The model is agnostic to where the tuned suppression might be originating from. Two possibilities that we considered are SSVEP gain or response amplitude (relative change in SSVEP power from baseline; Fig. 2d) and absolute SSVEP power (Fig. 2e). Could the differences across monkeys be due to differences in SSVEP power/gain? To test this, we plotted raw SSVEP amplitude, baseline amplitude and response amplitude (change in SSVEP amplitude from baseline) separately for the two monkeys (Fig. 7d). Interestingly, the raw SSVEP as well as the response amplitude had different shapes near 8 Hz for the two monkeys. In particular, the V-shape of the normalization strength for the 8 Hz condition in Monkey 2 (Fig. 7b, top) resembled a similar shape in the raw SSVEP and response amplitude in this monkey (Fig. 7d, right). Unfortunately, while this would explain the V-shape response at 8 Hz for Monkey 2, it cannot explain the 8 Hz responses in Monkey 1. Strength of normalization is likely to depend on several parameters in addition to the response amplitude, some of which are described in the Discussion.

## Discussion

Using multiscale recordings of MUA and LFP from macaque V1 together with scalp EEG, we have provided a definitive account of suppression acting along the TF axis, which modulates SSVEP responses differently depending on the relative TFs and orientations of the two driving stimuli. We also show that a tuned normalization model can account for most of the observations in our experimental data.

A previous study has looked at temporal frequency suppression in the spiking responses of cells in cat striate cortex using drifting gratings^30^, in which suppression was found to be tuned to higher temporal frequencies of the mask relative to the target, which appears to be different from the low-frequency tuned suppression observed here. Our SSVEP results are population neural responses dominated by synchronous activity due to counterphase stimulation, and thus not directly comparable to individual neurons’ spiking responses which were measured with drifting gratings optimized for each cell. In addition, since they only used the mask grating at the null orientation for each cell, they could not quantify the tuning of suppression as a function of relative mask orientation as we have done. Moreover, with their null gratings, they observed that suppression in spiking activity was tuned to higher temporal frequencies, whereas with our orthogonal gratings, we observed SSVEP suppression which was mostly untuned and symmetric. Overall, these differences suggest that LFP amplitude SSVEP responses may not be amenable to similar kind of suppression as observed in spiking responses.

We observed that different measures of neural activity have different SSVEP tuning (Experiment 1). There appear to be differences in the contrast response tuning of SSVEP in EEG vis-à-vis spikes and LFP, but we had insufficient number of stimulus repeats for EEG which limits any further inferences. However, LFP amplitude responses clearly preferred higher temporal frequencies than spiking responses, especially at higher contrasts. A simple explanation could be that LFP amplitude responses reflect afferent synaptic activity more than spiking activity, so our SSVEP tuning results are simply a reflection of the higher temporal frequency tuning of the lateral geniculate nucleus (LGN) as compared to V1^37^. More experiments are needed to further probe such tuning differences, which likely contribute to the overall temporal frequency tuning of the normalization pool and which our experiments do not address.

Our study builds on previous work on masking with human participants, many of which used similar normalization models^14–17^. One key difference is that while in many previous studies the test and mask TFs were fixed and mainly the contrast was varied^13,15,16^, here we have instead used a much larger number of TFs while keeping the contrast fixed. For modeling our results, we used a variant of the basic normalization model that was originally proposed to explain the spiking activity of individual neurons in the visual cortices^23,25^, but has been adapted since then to explain a lot of other phenomena, including population activity^27,45^. In the basic normalization model, the overall normalization strength depends on stimulus contrast but not on other properties such as orientation. The “tuned” normalization model used here relaxes this assumption^19,23,46^. These models have recently been used in attention studies in higher visual cortices^41–44^. We chose to make our normalization model tuned based largely on our results, with tuning in the joint TF and orientation space.

One practical implication of our results could be related to SSVEP studies of attention in human EEG which typically employ two or more stimuli, each flickering at a different TF. Specifically, paying attention to one stimulus will increase the overall normalization strength and reduce the amplitude of the remaining (unattended) SSVEPs, and this reduction will depend not only on attention but also on the flicker frequencies and properties of the attended and unattended stimuli. Reassuringly, though, our results suggest that if stimulus features (orientations in our case) are orthogonal, the interaction becomes non-specific, making the sensory component irrelevant for the most part.

We note that the stimulus-specific effects described here are likely to be important only in some cases. Often, the stimuli used in attention studies are small and occupy different locations in the visual field, either in the same or different hemifields^6,47–49^. It is unclear whether our results extend to these conditions, since the stimuli need to be spatially overlapping or at least be sufficiently close to each other to engage normalization mechanisms. Our results are more relevant to studies in which overlapping random dot patterns (RDPs) have been used, with different stimuli shown in different colors^7,9^. But even then, it is unclear whether the normalization observed with plaids is comparable to RDPs, and whether usage of different orientations is comparable to usage of different colors. We used oriented gratings and plaids since we were primarily looking at responses in V1 (which is driven well by such stimuli), which is one of the major contributors to the SSVEP^50^, and because considerable previous work has been done to characterize normalization using counterphasing plaid stimuli^14,17^. The receptive fields of V1 recording sites were anyway too small to allow two non-overlapping stimuli inside the RF. Overall, although we show suppression of SSVEPs at one frequency because of a competing tag at another frequency using plaid stimuli, these need to be further characterized using non-overlapping gratings within or across hemifields as well as RDPs in the EEG.

Orientation specificity of masking, whereby a parallel mask is more suppressive than an orthogonal mask has been shown before^15,17^. Since neurons preferring a particular orientation are spatially clustered in a pinwheel like structure in V1^51^, a normalization signal based on the pooled response of nearby neurons is likely to be stronger for a similar orientation than an orthogonal one, and hence more suppressive (this aspect is captured in a tuned normalization model). The TF specific suppression is more difficult to explain, since the detailed physiology of TF motifs in the primate is not clear; it is at best a representation of neuronal “islands” preferring high TFs scattered in a region which largely prefers low TFs^52^. A candidate mechanism is putative interactions between magnocellular and parvocellular pathways, which are known to have different temporal response characteristics^53–56^, although it remains unclear how these interactions lead to the TF profile that we observed.

In addition, the normalization signal may engage mechanisms stemming from sources other than TF tuning. For example, normalization strength may be obtained by integrating neural responses in a pool over some temporal integration window, akin to using a low-pass filter in the denominator of the normalization equation as suggested recently^16^. In this case, the normalization strength as a function of TF will depend on the characteristics of the low-pass filter. The IM components in the spike as well as SSVEP spectra offer further insights along these lines. Specifically, if the normalization pool oscillates with the driving frequency, the final response is of the form *sin(ωT)/(σ* + *sin(ωM) + sin(ωT))*, where the denominator is the normalization signal. Simple simulations of this form show that the resulting output spectrum has frequencies at many components, not just *ωT*, *ωM* and their differences/averages. However, in our data we only observed IM components at the differences/averages of *ωT* and *ωM*, which could be obtained using simple non-linearities such as squaring or taking the modulus. This suggests that the normalization signal may not oscillate at the driving frequencies, which could be achieved by low pass filtering the inputs before summing to generate the normalization signal. More experiments in which mask TFs are varied over a longer range are needed to tease out the components that contribute to the normalization pool.

Finally, another factor that would lead to stronger normalization at low frequencies and also explain the differences between spikes and SSVEP suppression profiles is the greater spatial summation for the LFP signal compared to MUA^57–59^. It is typically observed that coherence, which is a measure of phase consistency across electrode pairs, is highest at low frequencies and falls off with increasing frequency^60,61^. The phase difference across electrode pairs is typically zero^61^, suggesting that sinusoids comprising the LFP signal are in phase across space at low frequencies, but not at high frequencies. Therefore, even if the suppressive drive is independent of the TFs at the level of spiking activity, this drive could nonetheless be stronger at low frequencies when summed over a large space because of higher coherence.

While our model, like previous models of tuned normalization, provides a simple explanation of some of the observed results, there are a number of limitations. First, to fit normalization models to data, it is useful to have multiple contrast levels for both the target and mask stimuli, as done in previous studies^15,16^. Because we used a single contrast for both target and mask, we could not generate contrast response functions in Experiment 2, which could have enabled us to fit better normalization models to our results. Instead, we mainly aimed to determine the strength of the normalization signal that would explain our results and compared whether some of the candidate signals that may be contributing to the normalization pool (such as the response amplitude) varied with TF in a similar way as the normalization strength.

Second, the use of full-field stimuli to maximize the surface EEG SSVEP led to a drastic reduction in firing rates, such that we could not use spiking data to fit the normalization model, as done previously^23–25^. This suppression in firing rate has been observed in our previous studies as well^39,40^ in which we focused on gamma oscillations, because gamma oscillations (like SSVEP) are also most salient for large stimuli. Because the firing rates were low to start with, further suppression due to a mask, or changes in the phase relative to the stimulus^23^ were difficult to quantify for most of the cells. In addition, we could not record all stimulus conditions in the same session due to the large number of stimulus parameters and the animals’ unwillingness to perform the task for longer periods of time. Since chronic recordings like ours may not sample the same MUA across days (unlike the LFP which is much more stable), any attempt to compare MUA responses between different stimulus conditions must necessarily record them in the same session. Notwithstanding these limitations, we did fit the first stage of our normalization model to MUA responses (Experiment 1) and found that it accounted for the experimental data reasonably well.

We observed differences in temporal frequency tuning of MUA versus LFP, which raises the question whether a single, unified normalization model can generalize to different modalities. It is possible that our normalization model can also explain MUA response suppression in temporal frequency with additional constraints on the parameters, which we unfortunately cannot test. However, different neural measures can diverge considerably in response to the same stimulus, complicating the interpretation of the normalization model. In particular, the normalization model used here is unlikely to explain the SSVEP responses in EEG appropriately because this model does not capture neural synchrony, which can boost the EEG gain even when the underlying LFP power is negatively modulated^32^. Specifically, the tuning of this synchrony could potentially vary along the TF axis and differentially contribute to normalization strength, especially when observed at the level of EEG that is dominated by synchronous activity in the underlying population^31,33,62^.

Finally, our model cannot explain the differences in IM components for parallel versus non-parallel masks, which is likely due to the way non-linearities are applied to signals before being combined^15,17,38^. IM terms have previously proved to be useful in discriminating among computational models in two-input experiments^16,38,63^. Additionally, there is a strong expectation from previous literature that IM components could be temporally tuned given that they are sensitive to the orientations of the two component inputs^15,64,65^. In our data, we observed prominent peaks at |2F_TARGET_ − 2F_MASK_| in all orientation conditions except the parallel mask, but the amplitude of the difference IM components showed inconsistent trends. This happens because when the mask frequency is very close to the target frequency, the difference IM components are produced at low frequencies and which ride on the steep descent of the LFP power spectrum (where the power is already very high), making an accurate estimation of IM peaks difficult. This is a methodological limitation which is hard to overcome, unless one uses stimuli of much longer duration to get a finer frequency resolution or focuses on the higher-order IM peaks for which a larger number of trials would be needed. Furthermore, in order to compare different variants of normalization models (for example, as done by Foley^17^), stimuli must be presented at different contrast levels to effectively modulate both self and IM components. A more complete model, which explains SSVEP response generation in the time domain, is needed for capturing these aspects.

Temporal domain models have been used previously in the masking literature, but most of these studies relied on multiple contrast levels for the target or the mask for evaluating between candidate models^15–17^, something our study lacks. A recent study^29^ has used a delayed gain control model to explain the temporal structure of neural responses in various modalities of recording. Although they do not focus on temporal frequency steady-state responses, it seems conceivable that the impulse response function in their model formulation would relate to the observed temporal frequency tuning (Fig. 1) through the Fourier transform. Further, the time constant of the low-pass filter in the model could potentially explain some of the observed similarities between the temporal frequency tuning and the suppression due to the mask (Fig. 7). An investigation of these ideas merits further experiments with the mask frequency varied over much of the temporal frequency range, rather than in the immediate vicinity of the target frequency as we have done here.

In summary, here we have shown that SSVEP tags interact depending on both absolute and relative TFs as well as relative orientations. More data, using smaller stimuli, longer range of TFs, and multiple contrasts per TF, along with a detailed normalization model for SSVEP generation, are required to completely describe the biophysical mechanisms underlying TF specific normalization.

## Methods

The animal protocols in this study were reviewed and approved by the Institutional Animal Ethics Committee of the Indian Institute of Science and conducted in accordance with the guidelines approved by the Committee for the Purpose of Control and Supervision of Experiments on Animals.

### Recording setup

Recordings were performed on two female adult bonnet monkeys. Before training, a titanium headpost was implanted under general anesthesia. After the monkey learned the fixation task, a microelectrode array grid (Blackrock Microsystems) consisting of 96 active electrodes (1 mm long and 400 µm apart), was implanted in the right V1 cortex. The reference wires were either positioned securely under the dura of the site of craniotomy or wrapped around titanium screws nearby. Neural recordings were done using commercial hardware and software (Blackrock Microsystems), customized to record simultaneous scalp EEG in addition to LFP and multiunit activity (MUA). For recording EEG, we used Ag-AgCl cup electrodes (Grass) secured on the parieto-occipital scalp in the vicinity of the implanted array using conductive paste (Nuprep, Weaver and Company). We recorded EEG from up to 7 electrodes in Monkey 1 and 5 electrodes in Monkey 2; an EEG electrode placed near the headpost served as reference and another EEG electrode placed frontally was used as ground. Raw signals were band-pass filtered between 0.3–500 Hz and digitized at 2 kHz to obtain the LFP and EEG. Multiunits were extracted by filtering the raw signal between 250–7500 Hz followed by an amplitude threshold set at ~5 times the standard deviation of the signal. Multiunit waveforms were concurrently extracted and digitized at 30 kHz without any online sorting. More details of the surgery and recording setup can be found in our previous studies in which data (using different stimuli) from the same two monkeys were used^37,39,40^.

### Visual stimuli

Stimuli were presented on a gamma-corrected LCD monitor (BenQ XL2411Z) at a resolution of 1280×720 and 100 Hz refresh rate using custom software (Lablib, Mac OS X). Each experimental trial began with the presentation of a central white fixation spot (0.1° radius), which the monkey was required to fixate within a 2° window throughout the trial to get a juice reward. After a 1500 ms fixation delay, two stimuli with a duration of 1500 ms each were presented with an interstimulus interval (ISI) of 1500 ms, for a total trial length of 6 sec.

We ran a series of stimulus presentations in two different experiments in both monkeys. In Experiment 1, we presented full-field counterphase gratings (sinusoidal modulation, 50% duty cycle) at 6 different orientations (0°, 30°, 60°, 90°, 120° and 150°) at 5 contrasts (0%, 12.5%, 25%, 50% and 100%) and 8 TFs (0, 1, 2, 4, 8, 16, 32 and 50 Hz), with a spatial frequency of 4 cycles per degree (cpd). In Experiment 2, we presented plaid stimuli generated from a superposition of two oriented gratings with the same spatial frequency. Since the SSVEP amplitude response peaked in the ~8–16 Hz range in Experiment 1 (Fig. 1), we presented one grating of the plaid (“target” frequency) at either 16 Hz or 8 Hz, while the second grating varied in its frequency (“mask” frequency) around the target. At the 16 Hz target frequency, the mask frequency varied from 8 Hz to 24 Hz in steps of 2 Hz (n = 9), whereas at the 8 Hz target frequency, the mask frequency varied from 4 Hz to 12 Hz in steps of 1 Hz (n = 9). The orientation of the mask grating could be orthogonal to that of the target grating, or could differ from it in steps of 30°, resulting in 4 orientation conditions (parallel, 30° separation, 60° separation, orthogonal). The target frequency grating was fixed at one of the 6 orientations from Experiment 1 and presented at either 0% or 25% contrast (chosen pseudorandomly), whereas the mask frequency grating was always presented at 25% contrast, allowing us to record grating and plaid responses in the same session. However, note that in this configuration, grating response was obtained only for one (mask) orientation (when the target grating was at 0% contrast): the response for grating at the target orientation was obtained from a different session. For example, in Fig. 6, only null grating (orange trace) and plaid (black) were obtained in the same session, but the preferred grating (blue) was obtained from a different (usually adjacent) session. This did not seem to adversely affect our LFP analysis, since the LFP recorded at most sites remained stable over multiple days (with minor gain changes). However, it was difficult to meaningfully compare the SSVEP MUA amplitude between conditions; for instance, to analyze any effects of cross-orientation suppression^66^ of mask frequency on the target frequency. The MUA responses in our study were anyway highly suppressed because of the use of full field stimuli that were used to maximize the SSVEP responses in LFP and EEG.

### Electrode selection and data analysis

Data extraction and analyses were performed using in-house scripts written in MATLAB (The MathWorks, Inc.). As in our previous reports^37,39,40^, for all LFP analysis, we used only those electrodes that gave consistent LFP responses and stable estimates of receptive fields across days (Monkey 1: n = 65, Monkey 2: n = 39). The receptive field mapping procedure is explained in greater detail elsewhere^57,58^. Briefly, in each receptive field mapping session, we flashed small grating stimuli pseudorandomly at various locations over a rectangular window tiling the part of the visual field covering the aggregate receptive field of the microelectrode array, while the animal maintained steady fixation at a central white dot. Each stimulus was only briefly presented at one of four different orientations, and LFP responses to each position were averaged across orientations for every electrode. A two-dimensional Gaussian was fit to the LFP responses of each electrode to obtain estimates of the receptive field parameters (such as size and eccentricity) in each session, and electrodes which had unacceptable variability in their responses and fits across days were not used for further analysis.

For all analyses, we defined the analysis window as the 1-second period from 500 ms to 1500 ms after stimulus onset (a frequency resolution of 1 Hz, allowing us to examine the SSVEP response at all TFs used), with baseline defined as the corresponding period just before stimulus onset (−1000 ms to 0).

For generating the MUA plots in Experiment 1 (Fig. 1a,c and top panels in Fig. 1e,f), we picked across sessions only those LFP electrodes which had a signal-to-noise ratio (SNR^67^) of at least 2, accumulated at least 2000 spikes during the recording, and showed a firing rate increase above a predetermined cutoff (Monkey 1: 5 spikes/s, Monkey 2: 3 spikes/s) during the period from 500 ms to 1500 ms for the full contrast, static grating stimulus. Note that we used different cutoffs in the two monkeys because we observed that the spiking activity recorded in Monkey 2 was generally lower than that recorded in Monkey 1 (as in our previous report^37^, which used data from the same two monkeys in this study but a different microelectrode array in Monkey 1). This resulted in 20 and 12 MUA electrodes across sessions for the two monkeys.

To estimate the MUA response for these electrodes (top panels of Fig. 1e,f), we obtained the mean spike counts during the analysis window across all trials for each condition and subtracted the corresponding mean spike counts during baseline. For obtaining the summary statistics of MUA tuning (Fig. 1f, top panel), we normalized the spiking response of each MUA electrode by the maximum across conditions and then averaged across all MUA electrodes.

We obtained the peristimulus time histogram (PSTH) for a MUA electrode by binning spikes across each trial in 10 ms bins and averaging across trials for each condition. For visualization, we smoothed the PSTH using a 11-point Gaussian kernel with a standard deviation of 10 ms (Figs. 1a,c and 6b,e). For the MUA plots in Experiment 2 (Fig. 6), we selected only those multiunits which had a 2F spiking component (during the analysis window) of at least 5 spikes/s for the 16 Hz target frequency and 10 spikes/s for the 8 Hz target frequency in the plaid condition where the mask grating counterphased at the same frequency as the target grating. The firing rate spectra (Fig. 6c,f) were obtained by taking the absolute value of the Fourier transform of the raw, non-smoothed PSTH for each multiunit in the analysis window and averaging across multiunits.

To generate stimulus contrast profiles in Fig. 6, we first created sinusoids at the two stimulus TFs. We assumed that the spiking response is linear in the input followed by a nonlinearity [the Linear-Nonlinear (LN) model^25,68–70^], and simply full-rectified the sinusoids; this also doubled the frequency responses, modelling complex cell responses to counterphase gratings^25,71^. Applying rectification to the sinusoids before adding them generated the profile that most closely resembled the PSTH for the orthogonal plaid condition (Fig. 6a,d), whereas rectification after adding the sinusoids produced a profile that most closely resembled the PSTH for the parallel plaid condition (data not shown), consistent with an earlier model^17^.

To obtain an estimate of the LFP or EEG amplitude response (Fig. 1e,f, bottom panels; Figs. 2a; 5; 7a,d), we calculated S_ST_(2*f) - S_BL_(2*f), where f is the TF of the grating (Experiment 1) or the target/mask grating in a plaid (Experiment 2), and S_ST_(2*f) and S_BL_(2*f) are the magnitudes of the LFP or EEG amplitude spectrum at 2F during the analysis or baseline window respectively. We calculated the amplitude spectra S_ST_ and S_BL_ by Fourier-transforming individual trial data during the analysis or baseline window respectively and averaging the absolute value across trials. For summarizing the results of LFP or EEG amplitude tuning, we simply averaged the responses (bottom panel of Fig. 1f) or spectra (Figs. 3c and 4c) for the same orientation condition across electrodes and sets.

SSVEP responses are known to have fixed, idiosyncratic phase relationships with the stimulus, such that the signal-to-noise ratio of the SSVEP responses could, in theory, be improved by averaging the complex Fourier coefficients rather than their absolute values as we have done above. Reprocessing the SSVEP responses by taking the magnitude of the mean of Fourier-transformed data instead of the mean of the magnitude of Fourier-transformed data yielded similar results (data not shown).

We fit the SSVEP responses from Experiment 1 to a difference of exponentials function^36,37^, using an ordinary least-squares fit. We determined the preferred TF for each fit as the value of TF (resolution 0.1 Hz, maximum 100 Hz) at which the difference of exponentials peaked, allowing us to obtain a distribution of preferred TFs for each condition (Fig. 1e,f). If the preferred TF was less than 1 Hz, it was classified as low pass, otherwise it was classified as band-pass. The quality of fit was quantified as the fraction of total variance in the data explained by the fit^37^; the total variance was calculated by summing variances calculated separately for each contrast condition, such that values closer to 1 indicate very good fits to the data whereas values closer to 0 indicate poor fits.

For the tuning comparison of MUA and LFP (Fig. 1g, left), we used only those electrode/condition combinations across sessions in Experiment 1 which gave reliable estimates of both MUA and LFP amplitude tuning (fit quality ≥ 0.75) for band-pass electrodes (preferred TF ≥ 1 Hz). Since we used the same fit quality cutoff for all contrasts, there were in general more electrodes at higher contrasts since MUA/LFP tuning is expected to be noisier at lower contrasts. We also applied the same cutoffs as above for choosing MUA electrodes, to compare the tuning for a reduced set of electrodes (Fig. 1g, right). Note that in this tuning comparison, the LFP electrodes recorded on different days were treated as independent data points (without averaging across days), for a fair comparison with the MUA electrodes which were less stable and likely independent across days given our chronic recording setup. Nevertheless, we did fit LFP responses averaged across days and the resulting LFP tuning was qualitatively similar, except that we cannot meaningfully represent a comparison of LFP versus MUA tuning in a scatter plot (as in Fig. 1g).

Finally, we also averaged the preferred TF of the SSVEP responses separately for different contrasts in MUA, LFP and EEG, allowing us to compare tuning of SSVEP responses across scales (Fig. 1h).

### Experimental design and statistical analysis

Our experimental design was aimed at presenting counterphase gratings and plaids sampling the orientation space in V1, with multiscale recording of SSVEP. Experiments in humans recording scalp EEG commonly average data over tens to hundreds of stimulus presentations. However, due to the sheer number of conditions in our experiments, we restricted the number of stimulus presentations (~10 per condition per monkey), which yielded good LFP but noisier EEG SSVEP.

The tuning of MUA and LFP (Experiment 1) was compared using a paired, one-tailed Wilcoxon signed-rank test for the null hypothesis that the differences between preferred MUA TFs and preferred LFP TFs come from a zero-median distribution. Likewise, we compared the tuning of MUA and LFP separately for each monkey and contrast condition using a one-tailed Wilcoxon rank sum test for the null hypothesis that the preferred TFs of MUA and LFP come from distributions with equal medians.

To quantify the asymmetry of suppression (Experiment 2), we fit a linear regression model (MATLAB function *fitlm*) to the mean amplitude responses of the target frequency in each orientation condition separately for each monkey and each target frequency at 3 values of mask frequency both above and below the target frequency (Fig. 5; degrees of freedom = 4; slopes and associated significance levels from a *t*-test for zero slope are indicated). We did not use the farthest flanking conditions (8 and 24 Hz for the 16 Hz target, 4 and 12 Hz for the 8 Hz target) since they often contaminated the target frequency responses at even harmonics.

### Normalization model

We adapted the normalization model which has been extensively used and applied in the context of V1^25,26,72^. There are several variations of the basic normalization model; we chose the one by Carandini and colleagues^23^ which was originally put forward to explain many aspects of simple cell responses in macaque V1, including responses in the joint contrast and TF space as in our Experiment 1. We fit recorded data to the model in two stages.

In the first stage, we averaged the SSVEP responses for each electrode across sessions from Experiment 1 and fit the resultant mean amplitude responses for all non-zero contrast (*c*) and TF (*f*) conditions (4 contrasts × 7 TFs = 28 conditions) to the following function^23^:

1 $$R(c,f)={\left[{L}_{amp},(,f,),\frac{c}{\sqrt{\sigma {(f)}^{2}+{c}^{2}}}\right]}^{n}$$

where

2 $$\sigma {(f)}^{2}=\frac{1+{(2\pi f{\tau }_{0})}^{2}}{{({\tau }_{0}/{\tau }_{1})}^{2}-1}$$

The free parameters are the 7 amplitudes L_amp_ for each TF, time constants τ_0_ and τ_1_, and exponent *n*. The amplitude parameters L_amp_ characterize the TF tuning of the electrode at maximum contrast, and time constants τ_0_ and τ_1_ encapsulate the responsiveness of the neuronal population. We cannot explicitly attach any biophysical interpretation to these time constants or directly compare them to time constants obtained in other models of temporal response and gain control^16,29,73,74^, except noting that they may be thought of as “aggregate” measures of conductance at rest and at maximum contrast^23^. Finally, the exponent *n* captures any nonlinearities in the TF response generation process. We constrained all the model fit parameters to be non-negative; additionally, we constrained the exponent to be >1 (expansive nonlinearity). To compare tuning of SSVEP responses across scales, we fit responses from MUA, LFP and EEG electrodes separately. Note that we did not fit the phase responses as in the original model^23^, since phase advances could only be reliably observed for TFs around which most responses peaked (8 and 16 Hz).

In the second stage, we fit the data from Experiment 2 using model parameters recovered in the first stage to constrain the fits. The stimuli in Experiment 2 consisted of target and mask gratings at 25% contrast each (plaid condition), with orientations θ_TARGET_ and θ_MASK_ counterphasing at frequencies f_TARGET_ and f_MASK_ respectively, or only the mask grating at 25% contrast (grating condition) with orientation θ_MASK_, counterphasing at f_MASK_. To proceed, we make a key assumption that for a given electrode, the time constants τ_0_ and τ_1_ and exponent *n* remain constant across sessions, with only a possible change in the amplitude parameter L_amp_, due to potential differences in the orientation used or other recording conditions (such as noise levels). This assumption made it possible to “bridge” the fits across the data from Experiments 1 and 2 using a common set of parameters, as described below.

In the plaid condition, we aimed to characterize the suppression of the target frequency SSVEP due to the mask as a function of the difference in TF (δf = f_TARGET_ − f_MASK_) and the difference in orientation (δθ = |θ_TARGET_ − θ_MASK_ |). We expressed the target amplitude response R′(f_TARGET_) as a function of suppression arising from the mask using a variation of the plaid normalization model^23^ with the following equation:

3 $$R{\prime} ({f}_{TARGET}\,)={\left[{L}_{amp}({f}_{TARGET})\frac{{c}_{TARGET}}{\sqrt{\sigma {({f}_{TARGET})}^{2}+{{c}_{TARGET}}^{2}+S(\delta \theta ,\delta f){{c}_{MASK}}^{2}}}\right]}^{n}$$

where S(δθ, δf) captures the suppression originating from the mask, and σ(f) is as defined in Eq. (2). If c_MASK_ = 0 (grating condition) or if S(δθ, δf) = 0 (no suppression), Eq. (3) reduces to Eq. (1). Under these assumptions, plugging in time constants τ_0_ and τ_1_ and exponent *n* estimated from fits to Experiment 1 data (assumed to be the same for Experiments 1 and 2), allowed us to uniquely recover the amplitude parameter L_amp_(f_TARGET_) from amplitude responses obtained in the mask-only grating condition of Experiment 2.

Without assuming any functional form for S(δθ, δf), we first empirically estimated the overall magnitude of S(δθ, δf) from (3) by using the parameters obtained from the model fits in Experiment 1 as well as the amplitude L_amp_ recovered from the mask-only condition in Experiment 2. We normalized the empirical S(δθ, δf) using the maximum across conditions for each electrode and averaged across electrodes for each monkey (top row of Fig. 7b).

The linear regression fits to amplitude responses in different orientation conditions (Fig. 5) suggested that S(δθ, δf) can be modelled as a linear function of suppression originating from δθ and δf. Accordingly, we expressed this as:

4 $$S(\delta \theta ,\delta f)=\alpha +\beta ({f}_{MASK}-{f}_{TARGET}\,)$$

The parameter α is a measure of the mean “strength” of suppression acting on f_TARGET_ across all values of f_MASK_. Greater the value of α, more is the mean suppression. The parameter β is a measure of the “asymmetry” of suppression and has the effect of adjusting α to account for f_MASK_ relative to f_TARGET_. The sign of β controls whether suppression is greater for f_MASK_ > f_TARGET_ or f_MASK_ < f_TARGET_, whereas the magnitude of β controls the amount of this suppression. Small values of β close to 0 indicate that suppression is essentially independent of δf.

For each electrode, we averaged the TF amplitude response in the plaid condition across stimulus configurations having the same relative orientation (that is, averaging across different configurations of θ_TARGET_ and θ_MASK_ having the same δθ). We fit the resultant mean amplitude response to the Eqs. (3) and (4) for TF conditions f_TARGET_ ≠ f_MASK_ (three f_MASK_ frequencies each above and below f_TARGET_), using values of τ_0_, τ_1_ and *n* estimated from the fits to data from Experiment 1 and using L_amp_(f_TARGET_) estimated from the mask-only grating condition from Experiment 2. Values of f_MASK_ were in [10, 12, 14, 18, 20, 22] for f_TARGET_ = 16 Hz, and in [5, 6, 7, 9, 10, 11] for f_TARGET_ = 8 Hz. For each electrode and response condition, we estimated tuned suppression parameters α and β using ordinary least-squares fits, allowing α and β to vary over the entire range. Using estimated parameter values, we obtained the fitted S(δθ, δf) for each electrode and condition. To ascertain how the model behaves as a function of δθ and δf, we normalized S(δθ, δf), α and β separately using the maximum across conditions for each electrode and averaged across electrodes for each monkey [S(δθ, δf), bottom row of Fig. 7b; α and β, Fig. 7c].

We also fitted the model to the occipital EEG electrodes in the two monkeys and observed that it explained the EEG amplitude responses reasonably well (Supplementary Fig. S7). Although our model focusses on explaining SSVEP amplitude response modulation, we did fit the first stage of the model to the selected MUA electrodes (n = 32) from individual sessions of Experiment 1 and found that it accounted for the MUA responses equally well (Supplementary Fig. S7). However, we were unable to fit the full model to MUA responses from Experiment 2 due to a number of limitations. First, we used full-field stimuli to optimize the SSVEP amplitude in EEG which led to low MUA responses for gratings, and it was difficult to quantify further reduction in firing rates for plaids. Second, since we focused on the LFP, our plaid stimuli were not necessarily tuned to preferences of multiunits in a session. Finally, we could not always record MUA responses to all the target and mask combinations in the same session due to the large number of stimulus conditions or experimental constraints such as the animals’ unwillingness to do the task for longer durations. However, we do note that it is possible for TF normalization of spiking activity to be different from that of LFP or EEG.

## Supplementary information

Supplementary information.

## Data Availability

The data that support the findings of this study are available from the corresponding author upon reasonable request.
