# Supplementary material for: Interaction between steady-state visually evoked potentials at nearby flicker frequencies
Source: Sci Rep. 2020 Mar 24;10:5344. doi: 10.1038/s41598-020-62180-y (PMC7093459; doi:10.1038/s41598-020-62180-y)
Supplement: Supplementary file 1 — Supplementary information. [file 41598_2020_62180_MOESM1_ESM.pdf]

Supplementary Information

Interaction between steady-state visually evoked potentials at nearby flicker frequencies

Siddhesh Salelkar[1] and Supratim Ray[1,2*]

[1]IISc Mathematics Initiative, Department of Mathematics, Indian Institute of Science, Bangalore, India, 560012

[2]Centre for Neuroscience, Indian Institute of Science, Bangalore, India, 560012

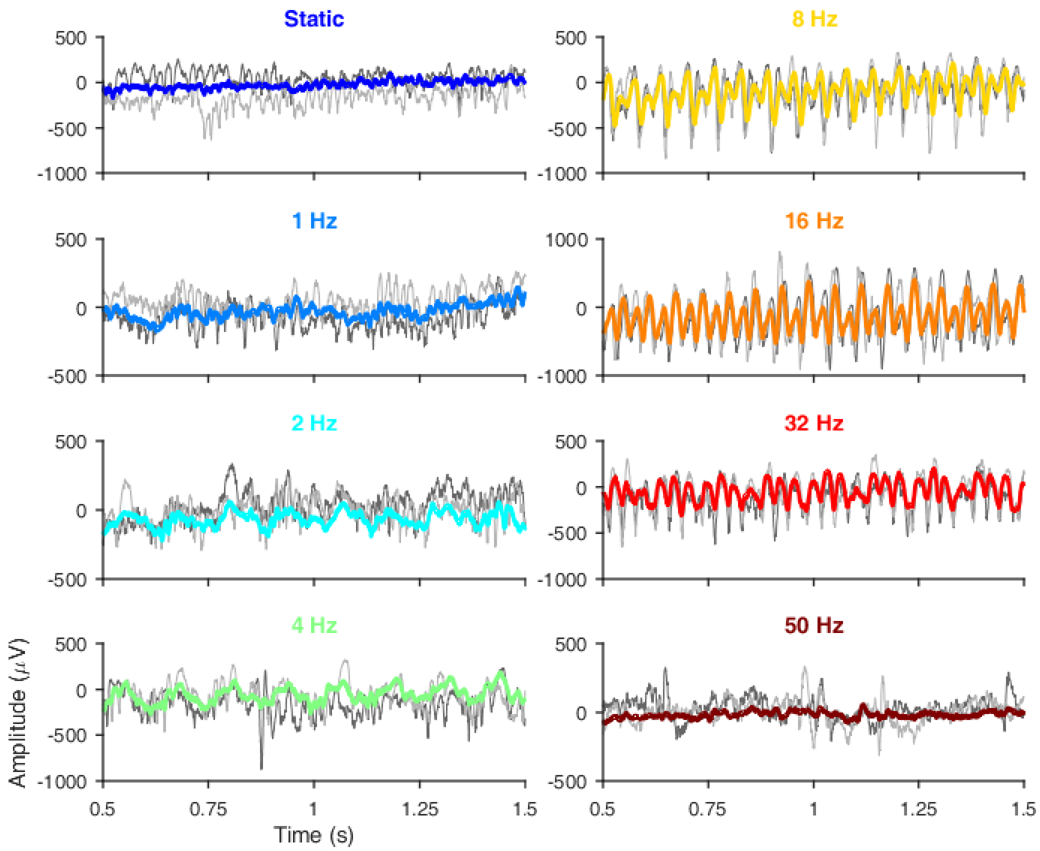

Supplementary Fig. S1. Magnified traces of SSVEP responses in LFP (Experiment 1)

SSVEP responses of an example LFP electrode (same as in Figure 1) during the analysis range from 500 ms to 1500 ms after stimulus onset. Colored traces represent the evoked response, whereas light and dark gray traces show the individual trials (same as in Figure 1).

Induced gamma oscillations can be observed in the individual traces at low TFs (up to 4 Hz). The y-axis of each panel has been scaled to the amplitude of its data for better visibility.

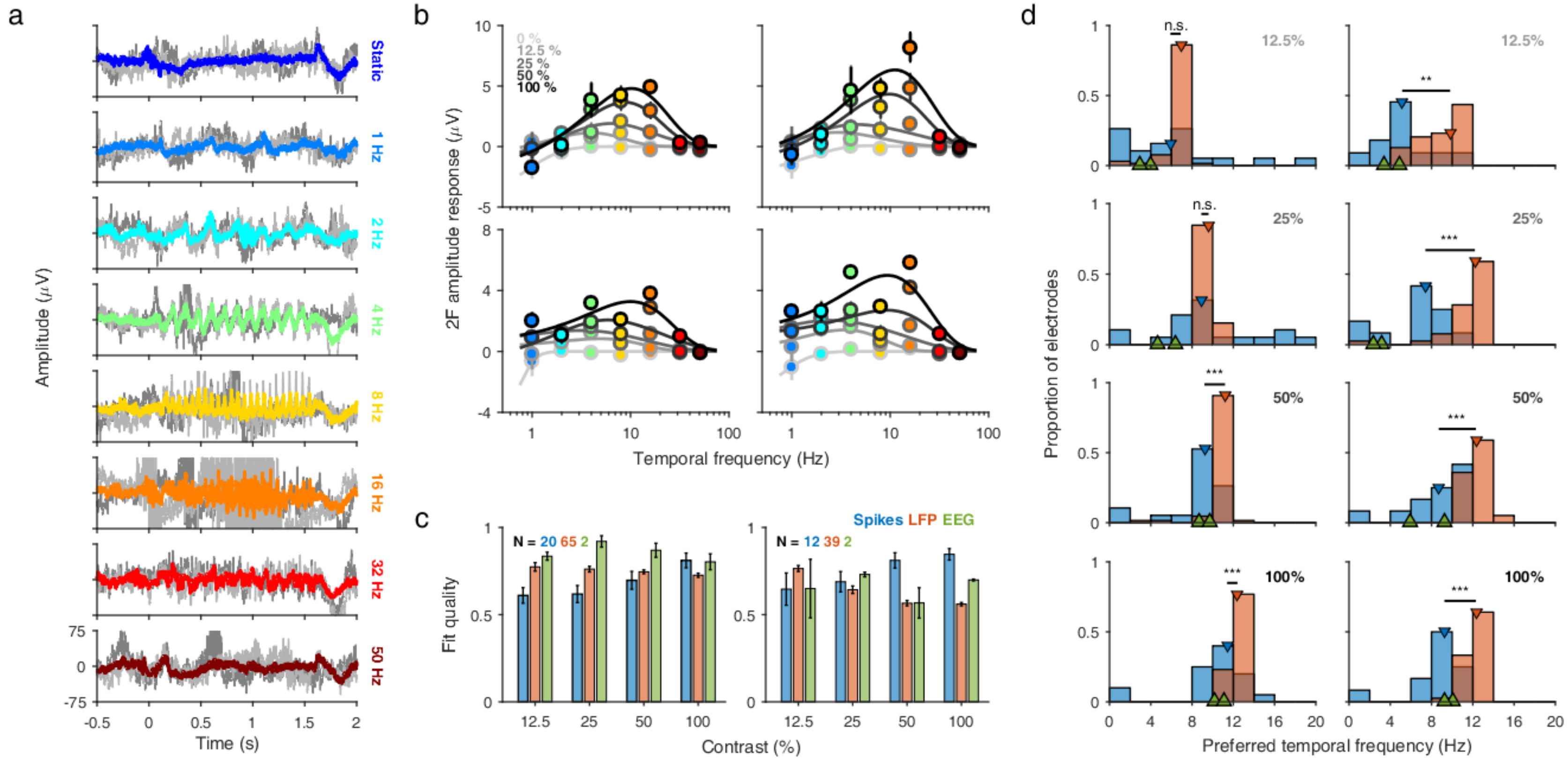

Supplementary Fig. S2. Comparison of MUA, LFP and EEG responses (Experiment 1)

(a) Evoked response (solid colored trace) of a simultaneously recorded occipital EEG electrode from the same session as the MUA/LFP electrode from Fig. 1a and 1b. The same two example trials for EEG (thin light and dark gray traces) are also plotted.

(b) Difference of exponentials fits (grayscale curves) to the mean amplitude response at 2F (colored circles) of the two occipital EEG electrodes (columns) for the two monkeys (rows). Fits were computed as a function of TF for each contrast separately. Colors as in (a). Error bars indicate ±1 SEM across sessions.

(c) Mean fit quality for the difference of exponentials fits obtained for MUA, LFP and EEG as a function of contrast for the two monkeys. Error bars indicate ±1 SEM across electrodes.

(d) Histograms showing the preferred TF of MUA and LFP electrodes as a function of contrast (rows) in the two monkeys (columns). Downward-pointing triangles indicate the median preferred TF for MUA and LFP. Upward pointing triangles indicate the preferred TF estimated for the two occipital EEG electrodes. Same color scheme as (c). Wilcoxon rank sum test for preferred MUA TF less than preferred LFP TF: ***, $p < 0.001$; **, $p < 0.01$; *, $p < 0.05$; n.s., not significant.

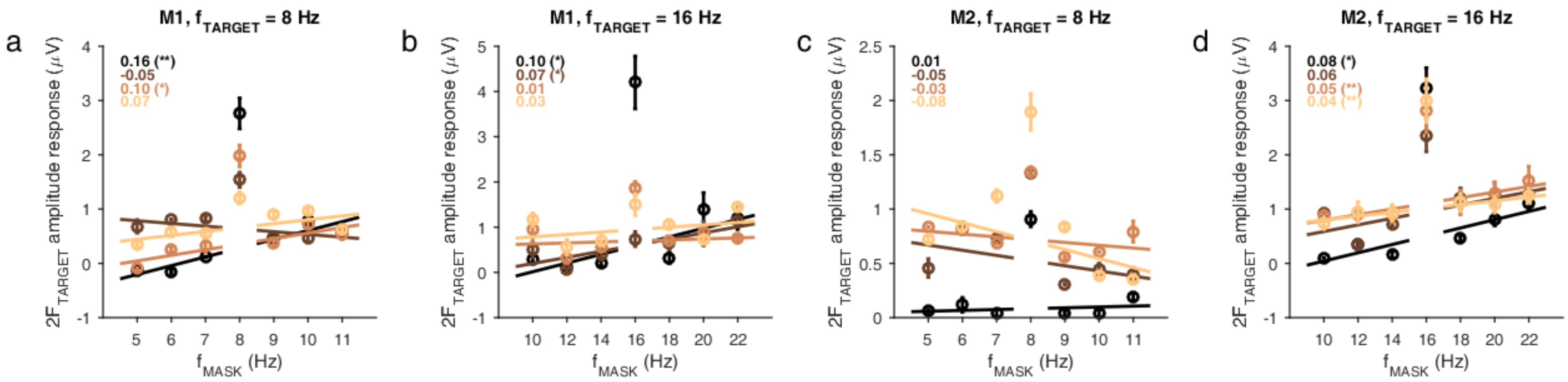

Supplementary Fig. S3. SSVEP amplitude suppression summary for EEG (individual monkeys)

Same as Fig. 5, but for EEG responses.

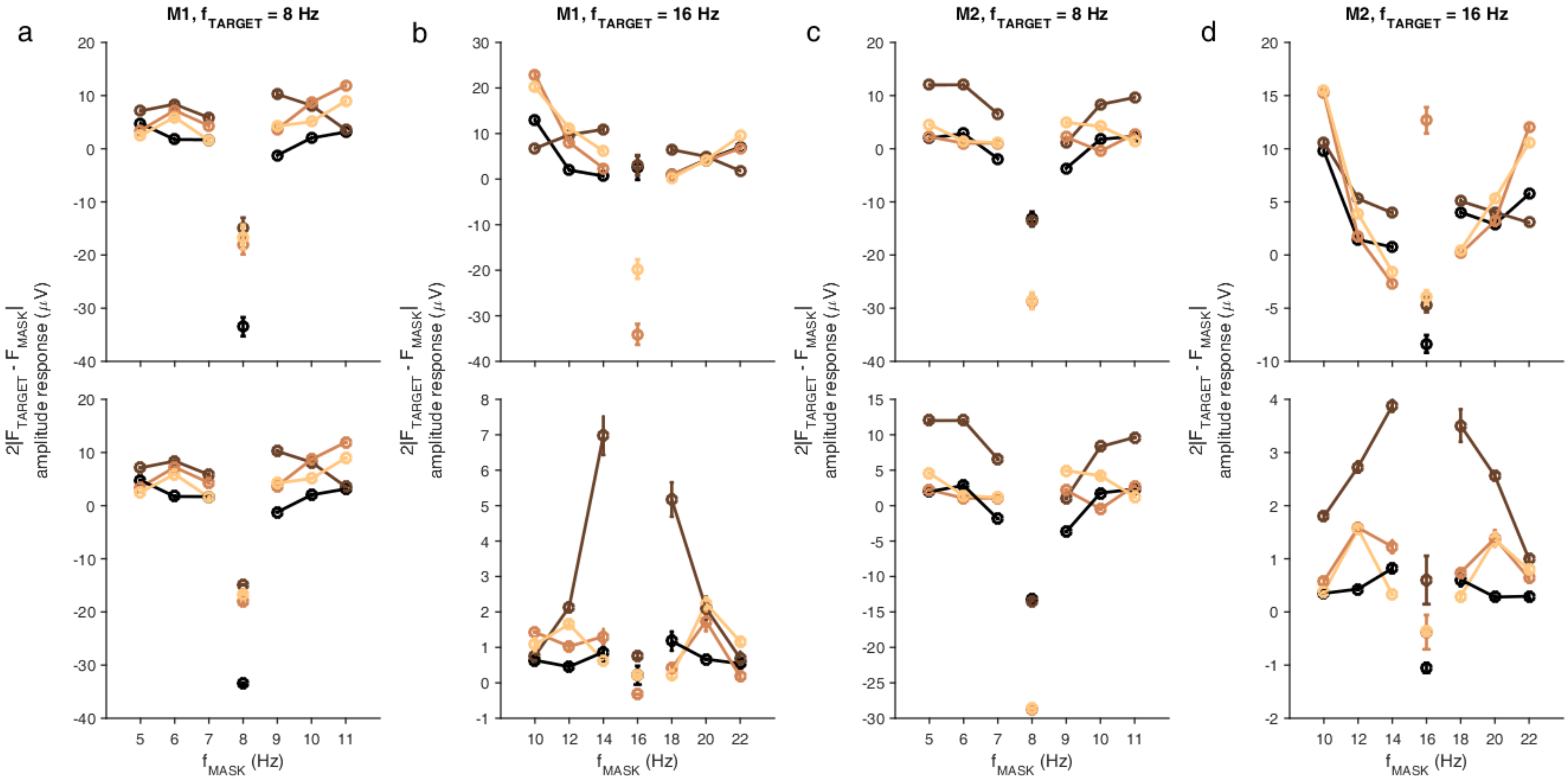

Supplementary Fig. S4. IM amplitude suppression summary

Same as Fig. 5, but for $|2F_{TARGET} – 2F_{MASK}|$ IM amplitudes. Linear regression fits are not plotted, since IM component responses for mask frequencies close to the target frequency could not be estimated properly (see Methods).

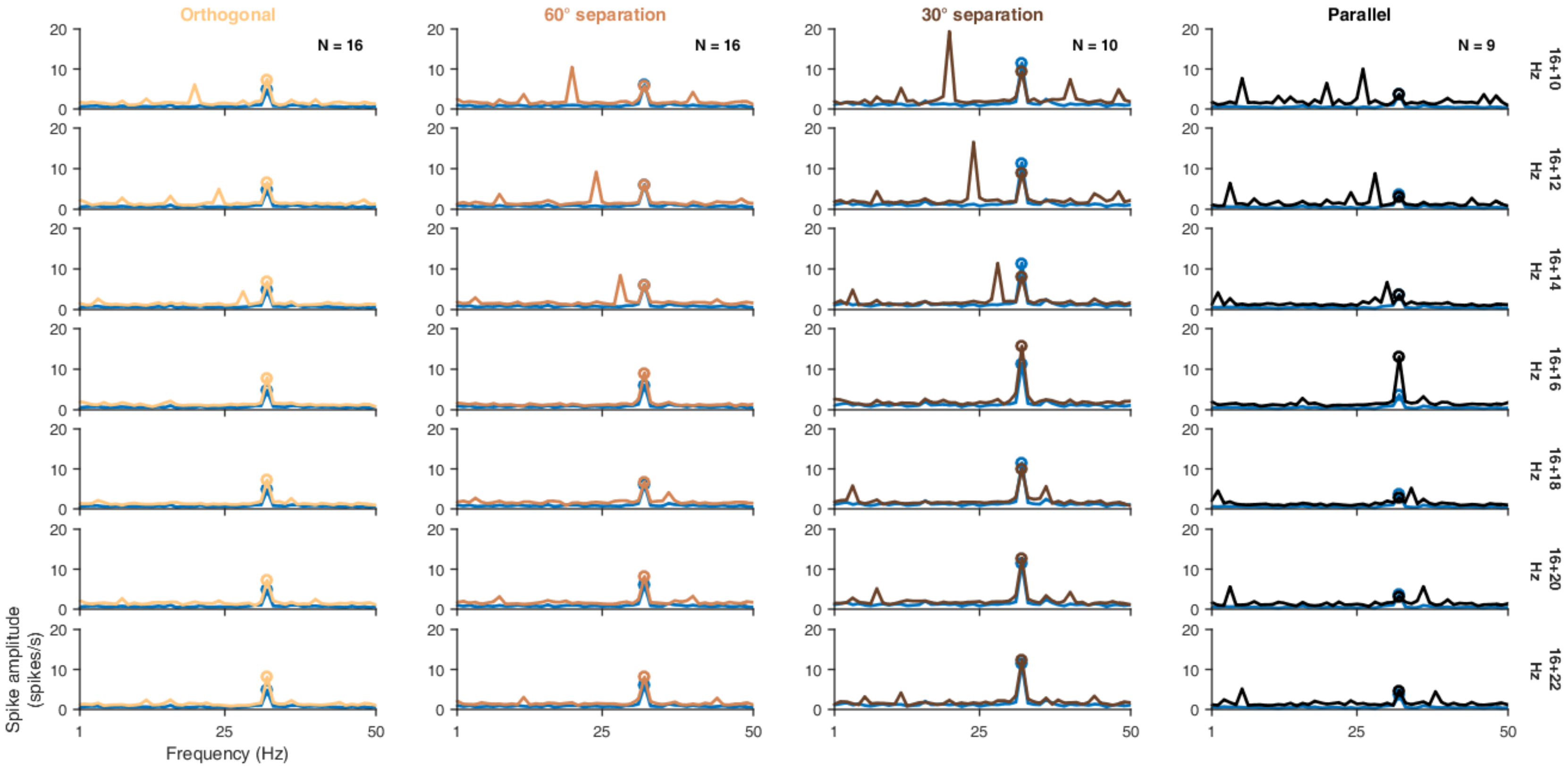

Supplementary Fig. S5. Spiking spectra (16 Hz target frequency)

Mean PSTH spectra of the population of good spiking electrodes across both monkeys for the 16 Hz target frequency, with rows showing different TF conditions and columns showing different orientation conditions as in Figs. 3 and 4. Spiking electrodes are selected using the same cutoffs as in Fig. 6, but without considering orientation selectivity. Grating-only PSTH spectra (blue) are averaged across the

same set of spiking electrodes, but from a nearby session, and are hence not directly comparable to the plaid spectra but plotted for representative purposes only.

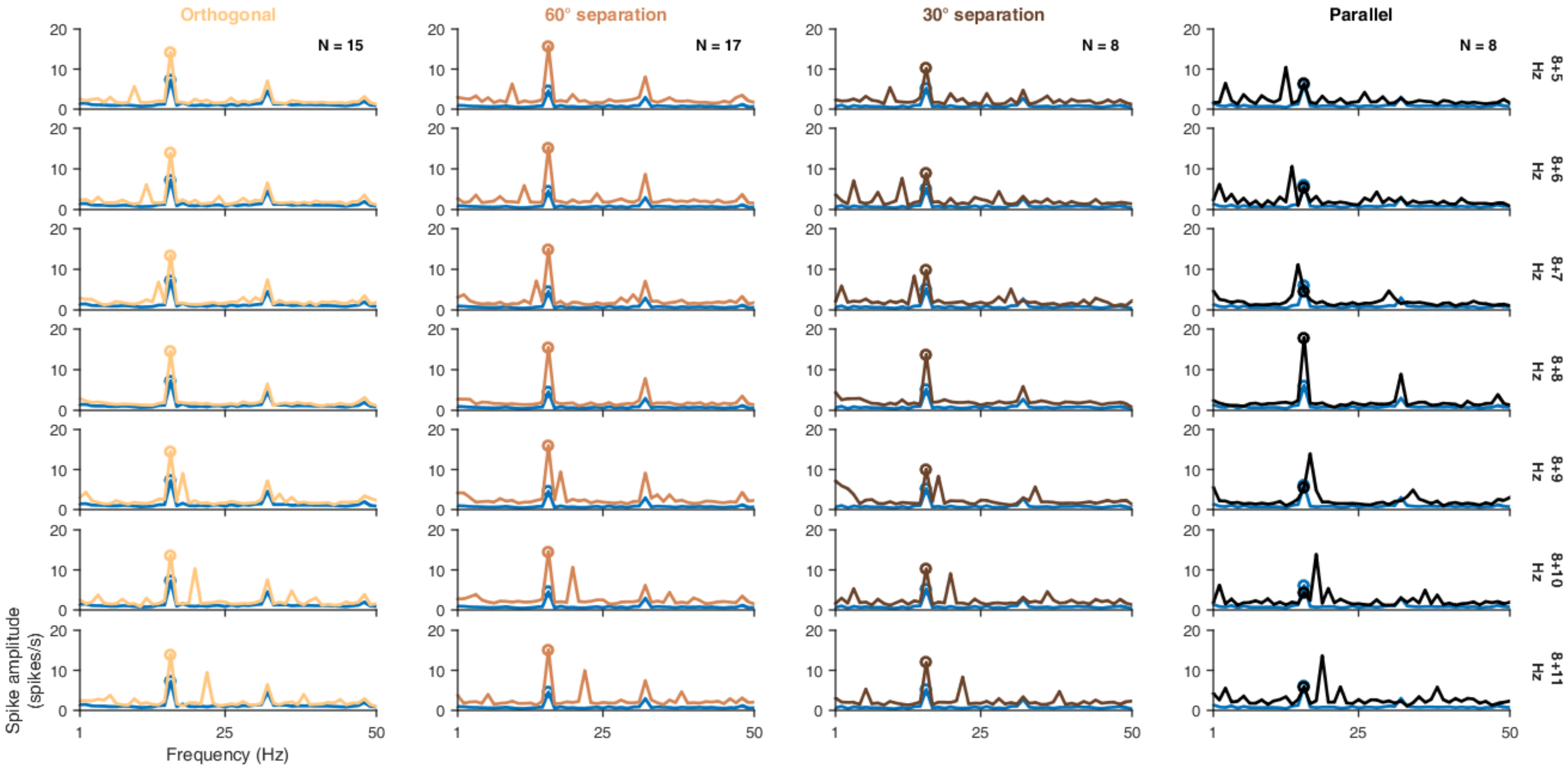

Supplementary Fig. S6. Spiking spectra (8 Hz target frequency)

Same as in Supplementary Fig. S3, but for the 8 Hz target frequency.

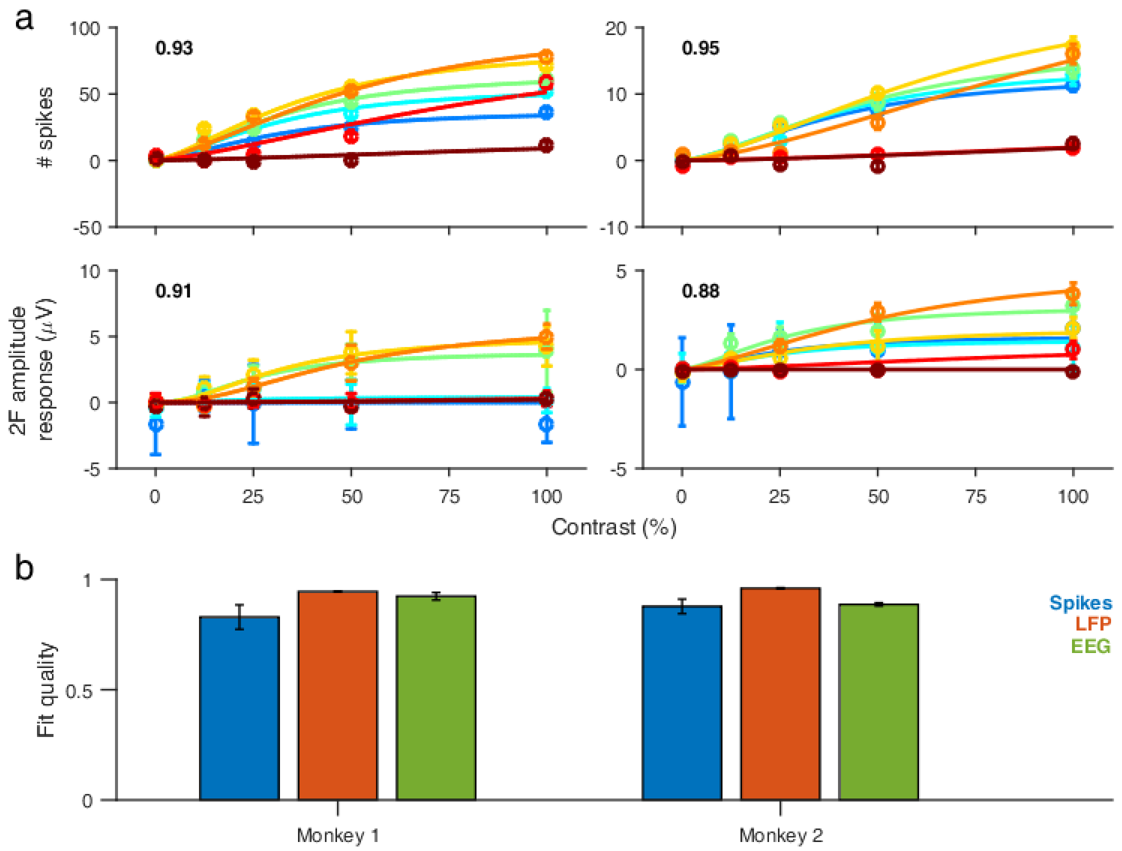

Supplementary Fig. S7. Normalization model fits

(a) Fits of the model (lines; Equations 1 and 2) to spiking responses (circles, top row) from two example MUA electrodes (left, Monkey 1, electrode 2; right, Monkey 2, electrode 50) and EEG amplitude response (circles, bottom row) from the left occipital EEG electrodes

from the two monkeys from Experiment 1. Colors as in Fig. 1. Inset number indicates model fit quality (the fraction of variance in the data captured by the fit). Error bars indicate ±1 SEM across sessions.

(b) Mean model fit quality to Experiment 1 data obtained for MUA, LFP and EEG electrodes for the two monkeys. Error bars indicate ±1 SEM across electrodes.
